# STEAP3 can predict the prognosis and shape the tumor microenvironment of clear cell renal cell carcinoma

**DOI:** 10.1186/s12885-022-10313-z

**Published:** 2022-11-23

**Authors:** Jiyue Wu, Qing Bi, Xiang Zheng, Huawei Cao, Changzhen Hao, Zejia Sun, Wei Wang

**Affiliations:** 1 Department of Urology, Beijing Chaoyang Hospital, Capital Medical University, 8 Gong Ti Nan Road, Beijing, 100020 China; 2 Institute of Urology, Capital Medical University, 8 Gong Ti Nan Road, Beijing, 100020 China

**Keywords:** STEAP3, ccRCC, Iron-metabolism, Prognostic, Tumor microenvironment

## Abstract

Clear cell renal cell carcinoma (ccRCC) is a common malignant tumor of the urinary system characterized by poor prognosis and difficult treatment. It has been reported that iron metabolism dysregulation is a common phenomenon in ccRCC and is closely related to the process of ccRCC. But still now, the exact function and underlying mechanisms of iron metabolism dysregulation in ccRCC have not been fully elucidated. In this study, we comprehensively investigated the prognostic value and potential role of STEAP3 (an iron metabolism-related gene) in ccRCC. STEAP3 is significantly up-regulated in ccRCC. High STEAP3 expression is associated with gender, hemoglobin level, pathological grade, tumor stage and significantly predicts an unfavorable prognosis of ccRCC patients. Functional enrichment analysis and evaluation of the tumor microenvironment indicated that STEAP3 was involved in the remodeling of tumor extracellular matrix and the shaping of an immune-suppressive tumor microenvironment to promote tumor metastasis and evade immune killing. Besides, the expression of STEAP3 is also associated with the expression of various immune checkpoint molecules and the IC50 of targeted drugs. Finally, we verified STEAP3 by RT-qPCR and IHC staining. In conclusion, we found that STEAP3 can serve as a candidate prognostic biomarker for ccRCC, and targeting STEAP3 and its biological processes may provide new references for the individualized treatment of ccRCC.

**Supplementary Information:**

The online version contains supplementary material available at 10.1186/s12885-022-10313-z.

## Introduction

Renal cell carcinoma (RCC) is the second most common malignancy of the urinary system, accounting for about 3% of all cancer diagnoses and deaths worldwide (1). According to statistics, there were 13,780 new deaths and 76,080 new cases of RCC in the United States in 2021, and these numbers will continue to increase (2). Renal cell carcinoma includes renal clear cell carcinoma (ccRCC), renal papillary cell carcinoma, renal chromophobe cell carcinoma and other subtypes, of which ccRCC is the most common subtype (about 75% ~ 80%) with the most invasive and the worst prognosis (3). It is estimated that approximately 25% of ccRCC patients have distant metastases such as lung, liver, and bone at the time of initial diagnosis (4). Surgical resection is still the main treatment for primary ccRCC patients as it is not sensitive to chemotherapy and radiotherapy (5). However, up to 40% of patients will recur after resection. Recently, the development of immune checkpoint inhibitors (ICIs) targeting cytotoxic T lymphocyte-associated antigen 4 (CTLA-4) and programmed death 1/programmed death ligand 1 (PD-1/PD-L1) raises new hope for cancer patients (6). Unfortunately, it is estimated that a small part of ccRCC patients can benefit from the treatment targeting ICIs (7). Therefore, it is imperative to explore novel prognostic biomarkers and potential therapeutic targets for ccRCC.

Iron is the most abundant trace element in the human body. It is involved in various cellular biological processes such as cellular respiration, energy metabolism, DNA replication, nucleic acid repair, and iron-dependent signal transduction (8, 9). Iron homeostasis is critical for normal cellular function and body homeostasis, and dysregulation of iron metabolism has been shown to be closely associated with liver cirrhosis, heart disease, diabetes, and cancer (10, 11). Specifically, iron overload has been found in many types of tumors including glioblastoma, lung cancer, ovarian cancer and breast cancer (12). As a kind of transition metal, excess iron can not only promote tumorigenesis, but also help tumor proliferation and distant metastasis through its pro-oxidative effects (13). The six-transmembrane epithelial antigen of prostate family member 3 (STEAP3) was first found in M1 cells of mouse bone marrow (14), which has the ferrireductase activity to reduce ferric iron (Fe3 +) to ferrous iron (Fe2 +) in endosomes (15). Recently, given the close relationship between STEAP3 and cellular iron homeostasis, its important role in a variety of cancers has been extensively studied. In uveal melanoma (UM), the expression of STEAP3 is significantly increased, and elevated STEAP3 was identified as a prognostic risk factor for UM (16). Han et al. found that STEAP3 is highly expressed in malignant gliomas with its expression negatively correlated with the overall survival (OS) of patients, and knockdown of STEAP3 in glioma can attenuate its aggressive phenotype (17). Furthermore, STEAP3 is found to be overexpressed in HCC cells, which promotes HCC progression by regulating EGFR and intracellular signaling (18). However, to our knowledge, there is no study to explore the role of STEAP3 in the progress and treatment of ccRCC.

In this study, by integrating information from multiple public databases, we found that STEAP3 is abnormally expressed in various types of cancer, and its abnormal expression is significantly related to the prognosis of cancer patients. As for ccRCC, we demonstrated that the expression levels of STEAP3 in tumor tissues were significantly higher compared with normal tissues, and its expression level was negatively correlated with the prognosis of ccRCC. Functional enrichment analysis revealed that STEAP3 may promote the growth, invasion, and metastasis of ccRCC by regulating the tumor microenvironment. Besides, we also explored the relationship between the expression level of STEAP3 and many immune checkpoints and targeted therapy sensitivity. Our results suggest that STEAP3 may be a potential prognostic biomarker and therapeutic target for ccRCC.

## Materials and methods

### Analysis of mRNA and protein expression levels of STEAP3

Firstly, we explored the mRNA expression level of STEAP3 in pan-cancer in the GENT2 database (http://gent2.appex.kr). GENT2 database integrates the gene expression profiles of more than 68,000 samples in the GEO database. Secondly, the RNA-seq data (TPM) and clinical information of 33 types of tumors and normal tissues in the TCGA database and the GTEx database were downloaded from the UCSC Xena database (http://xena.ucsc.edu/). The RNA-seq data were transformed into log2(TPM + 1) for subsequent analysis.

The mRNA expression levels of STEAP3 in 33 tumor and corresponding normal samples were compared, and paired comparison of tumor and adjacent tissues was performed in ccRCC. Four ccRCC datasets in the GEO database (including GSE36895, GSE53757, GSE15641, GSE10526) were used to further validate the mRNA expression levels of STEAP3 (Table 1). Besides, we also compared the STEAP3 protein expression in the CPTAC database and verified them with the immunohistochemical staining images in the HPA database.

**Table 1:** Information of 4 ccRCC datasets in the GEO database

Datasets	Sepecies	Sample size	References (PMID)
GSE36895	Homo sapiens	26 normal vs 26 ccRCC	22,683,710
GSE53757	Homo sapiens	72 normal vs 72 ccRCC	24,962,026
GSE15641	Homo sapiens	23 normal vs 32 ccRCC	16,115,910
GSE105261	Homo sapiens	9 normal vs 35 ccRCC	30,131,446

### Clinical correlation analysis

The”surviva”package in R was used for univariate cox regression analysis to explore the prognostic value of STEAP3 in pan-cancer. The survival indicators included OS (overall survival), DSS (disease-free survival), and PFI (progression-free interval). HR > 1 indicated that STEAP3 high expression is a risk factor for prognosis and *p* < 0.05 is considered statistically significant. According to the median expression level of STEAP3, ccRCC patients were divided into the low-expression group and the high-expression group. We then explored the survival differences between the two groups in the different clinical subgroups (age, gender, hemoglobin index, tumor location, pathological grade, clinical stage).

Besides, the association between the STEAP3 expression and clinicopathological parameters of ccRCC patients was also analyzed. To determine whether STEAP3 is an independent prognostic factor for ccRCC, we set the survival of ccRCC patients as the dependent variable, the expression level of STEAP3 and other clinical parameters as the predictive variables for univariate cox and multivariate cox regression analysis. According to the results of multivariate cox regression, we constructed a nomogram to guide clinicians perform prognostic analysis. The calibration plot was used to assess the performance of our nomogram.

### Potential mechanism analysis

Protein–protein interaction (PPI) network for STEAP3 was constructed in the STRING database (https://string-db.org/) with the minimum required interaction score set as 0.4. While the GeneMANIA database (http://www.genemania.org) was used to build the gene–gene interaction network for STEAP3. The "DESeq2″ package in the R was used to identify the differentially expressed gene (DEGs) between the two groups of ccRCC patients, the thresholds were set as |log2(FC)|> 1 and adj.*p* < 0.05. The”stat”package in R was used to identify the genes that were significantly correlated to STEAP3 in ccRCC, and | cor |> 0.3 and *p* < 0.05 were set as the threshold. The above two gene sets were integrated to perform GO and KEGG enrichment analysis to explore the potential biological functions of STEAP3 in ccRCC. GO terms included biological process (BP), cell composition (CC), and molecular function (MF). Besides, GSEA analysis was also conducted to further investigate the underlying mechanism of STEAP3. Enrichment analysis and GSEA analysis were implemented through the "ClusterProfiler" package in R (19).

### Assessment of tumor microenvironment and immune infiltration

TIMER2.0 is an interactive portal for comprehensive analysis of the infiltration levels of different immune cells in the tumor microenvironment. Here, we investigated the correlation between the STEAP3 expression and the infiltrating abundance of multiple immune cells (B cells, NK cells, macrophages, CD8 + T cells, CD4 + T cells, CAFs, and MDSCs) in ccRCC. Besides, the "Gene_Corr" module of TIMER2.0 was also used to explore the correlation between STEAP3 and a variety of immune cell marker genes, *p* < 0.05 was considered statistically significant. Validation of correlation analysis was performed by the GEPIA database.

### Analysis of the correlation of Immune checkpoints and the sensitivity of targeted drugs

To provide guidance on the treatment of ccRCC, we explored the correlation between STEAP3 and immune checkpoints. The immune checkpoints were integrated from several published literature. Besides, we also obtained information of ccRCC patients receiving targeted therapy in the TCGA database to analyze the relationship between the outcomes of targeted therapy and the expression level of STEAP3 expression in tumor tissues. The drug susceptibility test data of 60 cancer cells were obtained from the CellMiner database to screen potentially effective targeted therapy drugs for ccRCC, Pearson correlation analysis was performed to explore the correlation between the expression level of STEAP3 and the sensitivity of targeted therapy drug, with |R|> 0.3 and *p* < 0.05 as the threshold.

### Cell cultures and mRNA content determination

All cells were purchased from the American Type Culture Collection (ATCC). The human renal cortex proximal tubule epithelial cells (HK-2 cells) were maintained in DMEM/F12 medium (Gibco, USA), the human skin metastasis-derived clear cell renal cell carcinoma cells (Caki-1) and the human primary clear cell adenocarcinoma cells (786O cells) were cultured in McCoy’s 5A Medium (iCell, China) and RPMI 1640 medium (Gibco, USA), respectively. All mediums were supplemented with 10% fetal bovine serum (Gibco, USA), and cells were cultured at 37 °C and 5% CO2.

The Total RNA Isolation Kit (Vazyme, China) was used to isolate the total RNA according to the manufacturer’s instructions. Using the HiScript III RT SuperMix for qPCR (+ gDNA wiper) (Vazyme, China) to conduct the reverse transcription and the qPCR were accomplished by the AceQ Universal SYBR qPCR Master Mix (Vazyme, China). The comparative Ct method was used for evaluating the relative expression levels of STEAP3. Primer sequences of STEAP3 and GAPDH for RT-qPCR was shown in Table 2.

**Table 2:** Primer sequences of STEAP3 and GAPDH for RT-qPCR

Gene		Sequence (5’- 3’)
STEAP3	F^a^	CCAATGCTGAGTACCTGGC
	R^a^	ATCTCCGAGACAGCACGC
GAPDH	F	GCCTTCCGTGTCCCCACTGC
	R	GGCTGGTGGTCCAGGGGTCT

^a^*F* forward, *R* reverse

### Tissue Microarray and Immunohistochemistry (IHC)

*Tissue microarray was obtained from Outdo Biotech Co., Ltd. (Shanghai*, China), which included 40 ccRCC lesions and 40 adjacent normal tissues. The tissue microarray was treated to antigen retrieval with EDTA buffer and then incubated overnight with rabbit antihuman polyclonal STEAP3 antibody (1:400, Bioss, Beijing, China) at 4 °C. Next, the tissue microarray was incubated with the secondary antibody for 30–40 min and observed under an optical microscope. IHC staining results were scored by a pathologist blinded to sample classification.

### Statistical analysis

R software (version 3.6.3) was used to perfrom all statistical analyses in this study. Differentially expressed genes between tumor tissues and normal tissues were assessed by t-test, and Kaplan–Meier analysis was used to explore the prognosis of cancer patients The correlation between the STEAP3 expression and clinicopathological parameters was analyzed by Chi-square test, Fisher's exact test, and Rank sum test. All statistical tests were two-sided and P < 0.05 was considered statistically significant.

## Results

### The expression level of STEAP3 in pan-cancer and ccRCC

**Fig. 1:**
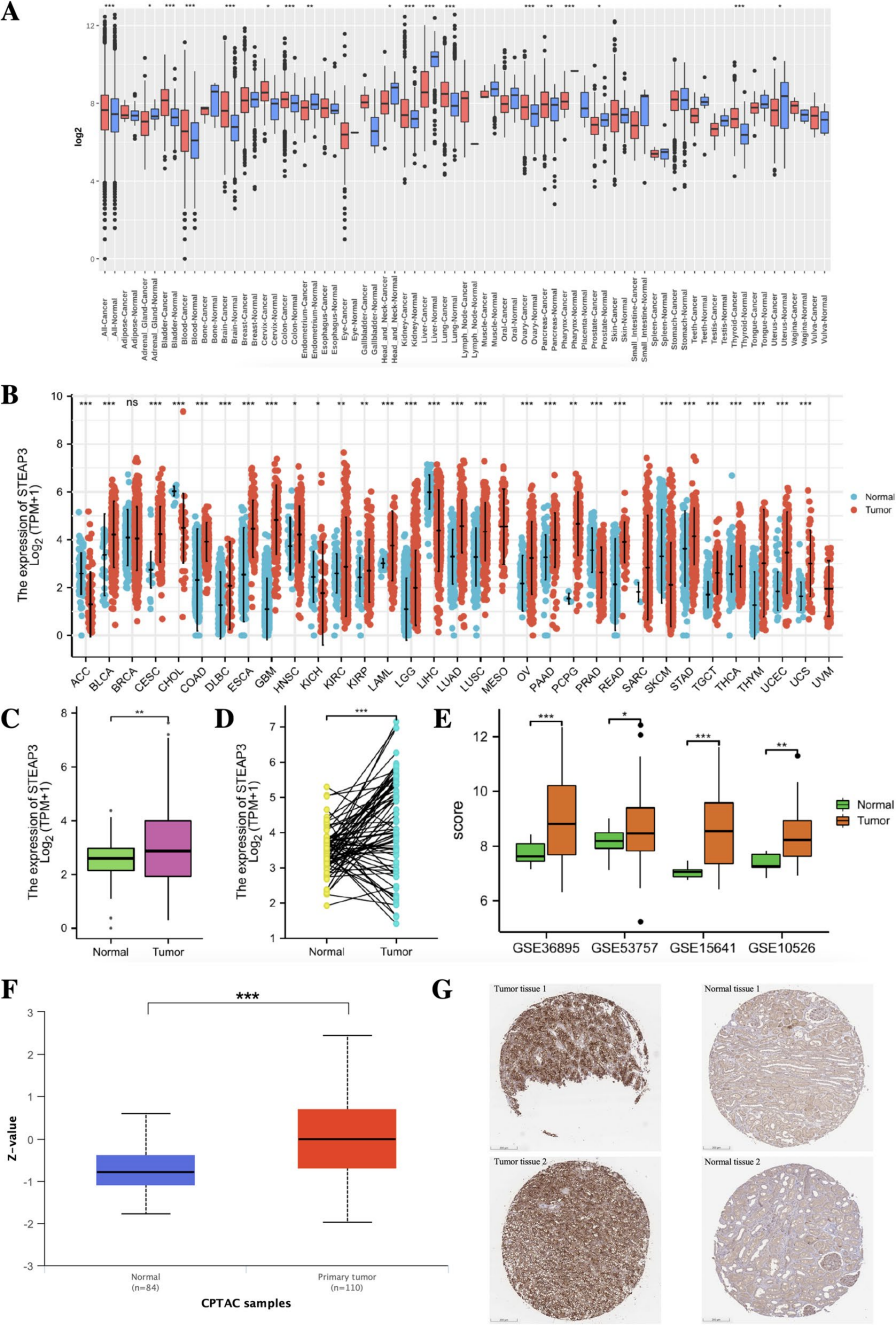
The expression level of STEAP3 in pan-cancer and ccRCC. **A** STEAP3 mRNA expression in pan-cancer, compared with normal tissues in the GENT2 database. **B** STEAP3 mRNA expression in pan-cancer, compared with normal tissues in the TCGA and GTEx databases. **C** Analysis of STEAP3 mRNA expression in ccRCC and normal tissues in the TCGA database. **D** STEAP3 mRNA expression in 72 pairs of ccRCC tissues and adjacent normal tissues. **E** STEAP3 mRNA expression in ccRCC across four GEO datasets. **F** The protein expression level of STEAP3 in ccRCC and normal tissues in the CPTAC database. **G** Immunohistochemical staining of STEAP3 from the HPA database (Two ccRCC tissues vs two normal tissues). *p < 0.05; **p < 0.01; ***p < 0.001

We analyzed the expression profile of STEAP3 in pan-cancer through the GENT2 database. The results showed that compared with corresponding normal tissues, STEAP3 was up-regulated in bladder cancer, blood cancer, brain cancer, cervix cancer, colon cancer, kidney cancer, lung cancer, ovary cancer, pancreas cancer and thyroid cancer tissues. While it was down-regulated in adrenal cancer, endometrium cancer, head and neck cancer, liver cancer, pharynx cancer, prostate cancer and uterus cancer tissues (Fig. 1A). The RNA-seq data obtained from the UCSC database showed that the mRNA expression level of STEAP3 was abnormally expressed in 29 types of tumor tissues (The text labels on the x-axis represent the abbreviations of 33 tumors in the TCGA database, of which KIRC represents the ccRCC), and most of the results were consistent with that in the GENT2 database (Fig. 1B).

In ccRCC, we found that the mRNA expression level of STEAP3 in tumor tissues was higher compared with normal tissues (Fig. 1C), and paired comparison also indicated that the mRNA expression in tumor tissues was significantly higher than in adjacent tissues (Fig. 1D). Four ccRCC datasets including GSE36895, GSE53757, GSE15641 and GSE10526 from the GEO database further validated the above results (Fig. 1E). The data from the CPTAC database showed that the protein expression level of STEAP3 was also significantly higher in tumor tissues (Fig. 1F). The above finding in the CPTAC database was further supported and confirmed by the results of immunohistochemical staining against STEAP3 in the HPA database (Fig. 1G). It can be seen that STEAP3 was abnormally expressed in various tumor tissues, and its mRNA and protein expression levels in ccRCC tissues were significantly higher than those in normal tissues, which indicated that it may promote the process of ccRCC.

### Prognostic significance of STEAP3 in pan-cancer and ccRCC

**Fig. 2:**
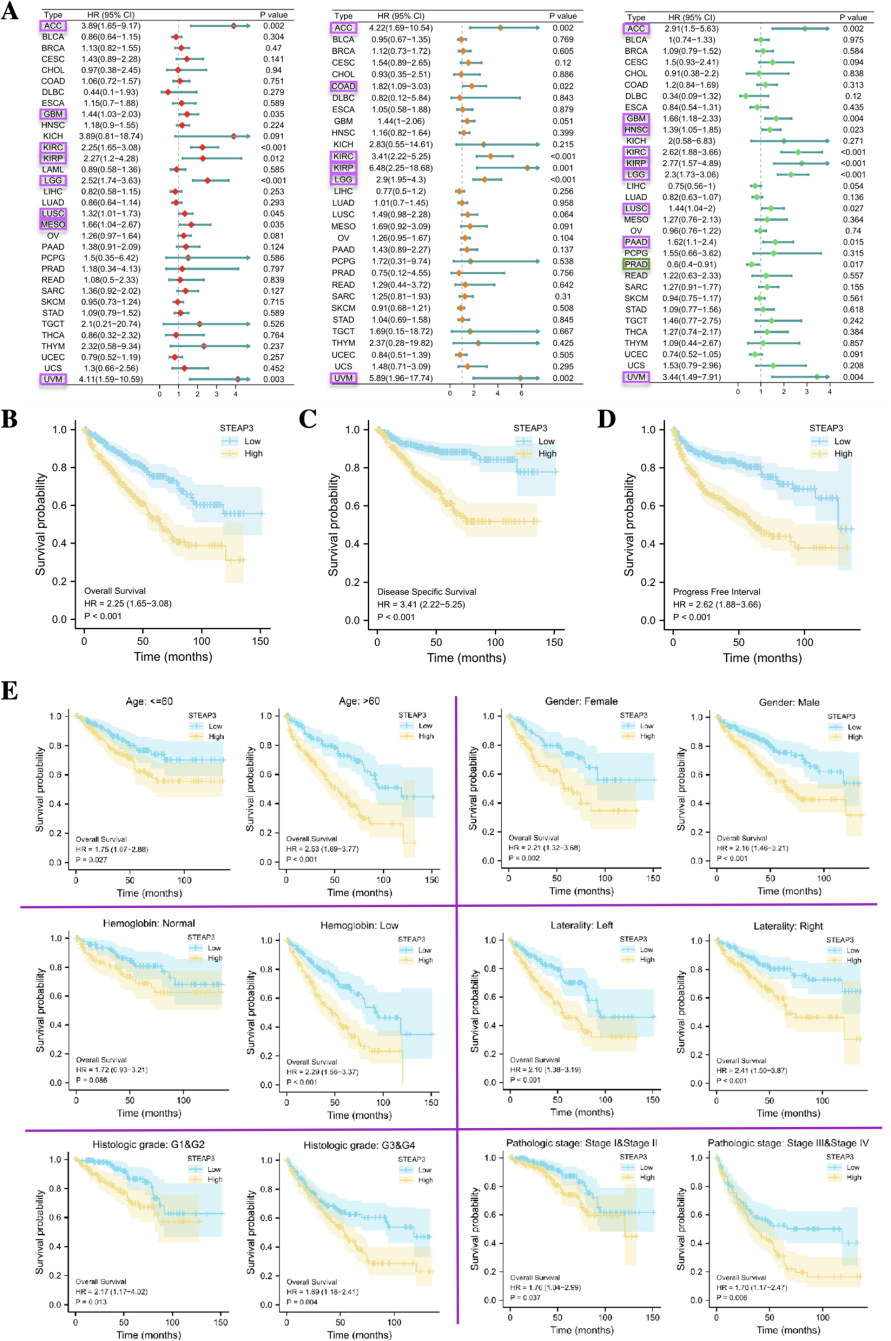
Prognostic significance of STEAP3 in pan-cancer and ccRCC. **A**-**C** The forest plots of univariate Cox regression analyses (OS, DSS, PFI). **D** K-M survival analysis comparing STEAP3 low- and high-expression ccRCC patients. **E** K-M survival analysis between the two groups in different clinical subgroups

By integrating the clinical information of ccRCC patients, we investigated the effect of STEAP3 expression on the prognosis of pan-cancer. Forest plots showed that high expression of STEAP3 was associated with poor OS in ACC, GBM, KIRC, KIRP, LGG, LUSC, MESO, and UVM (Fig. 2A), and the high expression of STEAP3 in ACC, COAD, KIRC, KIRP, LGG and UVM was significantly negative correlated with DSS (Fig. 2B). As for PFI, the plots showed that high expression of STEAP3 may be a prognostic risk factor for ACC, GBM, HNSC, KIRC, KIRP, LGG, LUSC, PAAD and UVM, while may be a protective factor for PRAD (Fig. 2C).

For ccRCC patients, survival analysis showed that OS, DSS, and PFI of patients with higher STEAP3 expression were worse than those with lower STEAP3 expression (Fig. 2D). For patients in different clinical subgroups (age, gender, hemoglobin index, tumor location, pathological gradeand clinical stage), K-M survival analysis also indicated that the survival of the patients with higher STEAP3 expression was significantly shorter regardless of the subgroup the patients belonged to (Fig. 2E). This indicated that the expression level of STEAP3 can predict the prognosis of ccRCC patients, and patients with higher STEAP3 expression may have a worse prognosis.

### Construction of prognostic nomogram

**Fig. 3:**
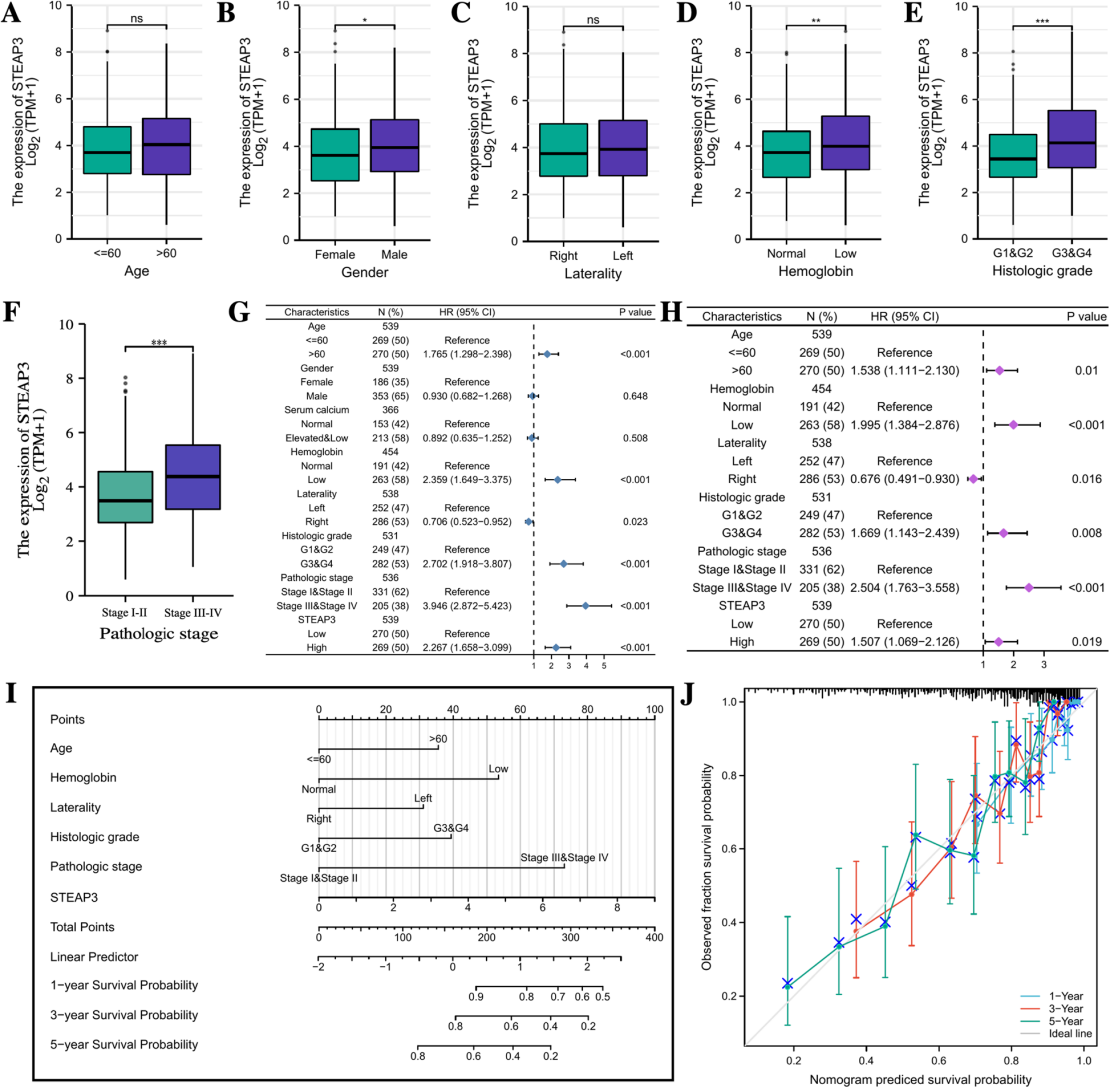
Clinical correlation analysis and construction of the nomogram. **A**-**F** Box plots showing the STEAP3 expression among different clinical subgroups. **G** Forrest plot of univariable Cox regression analysis in ccRCC. **H** Forrest plot of multivariable Cox regression analysis in ccRCC. **I** Nomogram integrated STEAP3, age, hemoglobin, laterality, histologic grade and pathologic stage. **J** Calibration plot of the nomogram at 1, 3 and 5 years

We further investigated the realationship between the expression level of STEAP3 and clinical parameters of ccRCC patients. There were no significant differences in STEAP3 expression among the subgroups of age (> 60 vs <  = 60, according to the World Health Organization's recommendations, people over the age of 60 in developing countries can be defined as the elderly), and tumor location (left kidney vs right kidney) (Fig. 3A, 3C). While in the subgroups of gender (male vs female), hemoglobin index (normal vs low), pathological grade (G1-2 vs G3-4) and clinical stage (Stage I-II vs Stage III-IV), we found that female patients with low hemoglobin had higher STEAP3 expression (Fig. 3B, 3D) and patients with higher pathological grades and clinical stages also had higher STEAP3 expression (Fig. 3E-F).

To determine whether the expression level of STEAP3 is an independent prognostic factor for ccRCC patients, we performed univariate Cox and multivariate Cox regression analyses. The results showed that STEAP3 was an independent prognostic factor for ccRCC. Besides, advanced age, lower hemoglobin level, higher pathological grade and clinical stage were also considered prognostic risk factors for ccRCC, while lesion in the right kidney was considered a protective factor (Fig. 3G-H). By integrating the above prognostic factors of ccRCC, we constructed a nomogram that could provide clinicians with accurate prognostic information. Clinicians can use our nomogram to score ccRCC patients based on clinical parameters and STEAP3 expression to estimate their survival at 1 year, 3 year and 5 years (Fig. 3I). The calibration plot showed that the predicted results based on STEAP3 in 1 year, 3 years and 5 years showed good consistency with the actual results (Fig. 3J).

### Identification of genes and proteins interacting with STEAP3

**Fig. 4:**
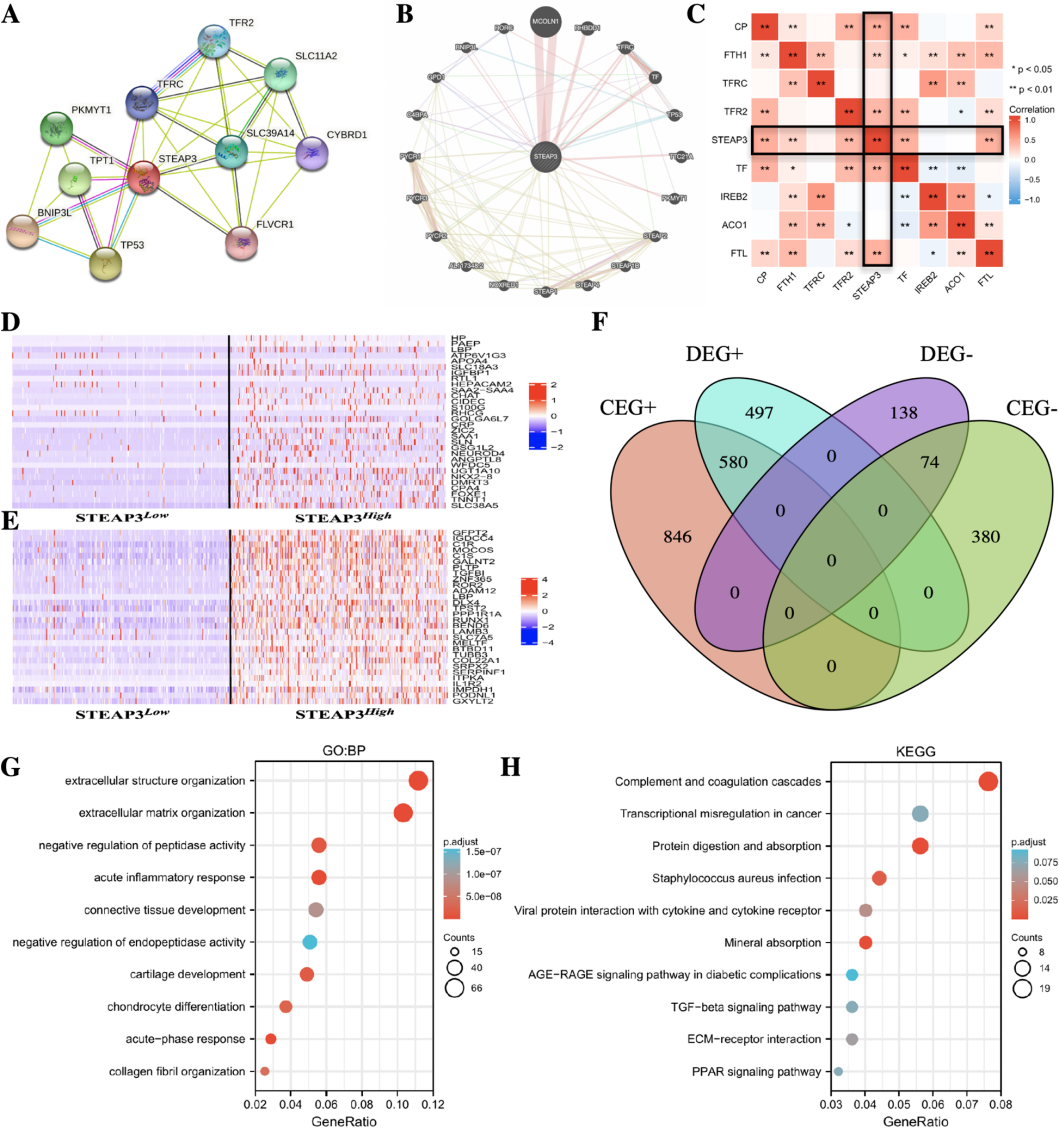
Potential Mechanism of STEAP3. **A** The PPI network of STEAP3. **B** The gene–gene interaction network of STEAP3. **C** The correlation matrix shows the correlations between STEAP3 and iron metabolism-related genes in ccRCC. **D** Heatmap of the top 30 DEGs between the two groups. **E** Heatmap of the top 30 CEGs associated with STEAP3. **F** Venn diagram shows genes closely related to STEAP3 in ccRCC. **G** Top 10 enrichment terms in BP categories of the STEAP3-related genes. **H** Top 10 KEGG enrichment pathways of the STEAP3-related genes

The PPI network of STEAP3 contained 31 interactions and 11 nodes (including TFRC, TFR2, SLC11A2, etc.) (Fig. 4A). The gene–gene interaction network of STEAP3 showed the 20 genes most related to STEAP3, including TFRC, TF, TP53, etc. (Fig. 4B). Since most of these genes or proteins were involved in iron metabolism, we further explored the correlation between STEAP3 and iron metabolism-related genes. The results showed that in ccRCC tissues, the STEAP3 expression was significantly positively correlated with FTL, TF, TFR2, FTH1, and CP (Fig. 4C), indicating that STEAP3 was likely to be closely related to the regulation of iron metabolism in ccRCC.

### Potential mechanism of STEAP3 in ccRCC

DEGs and co-expressed genes (CEGs) between the high-expression group and the low-expression group were identified using RNA-seq data obtained from the TCGA database. Figure 4D showed the top 30 DEGs between the two groups, while Fig. 4E showed the top 30 CEGs associated with STEAP3. By intersecting the DEGs (log2FC > 1) with CEGs (cor > 0.3) and DEGs (log2FC < 1) with CEGs (cor < 0.3), a total of 654 genes that were closely related to STEAP3 in ccRCC were obtained (Fig. 4F). KEGG and GO enrichment analysis was performed on these 654 associated genes to explore the potential biological functions of STEAP3 in ccRCC (Supplementary Table 1–2). GO annotation showed that these genes were mainly enriched in the extracellular structure organization, extracellular matrix organization, connective tissue development, and collagen fibril organization, etc. (Fig. 4G). KEGG pathway enrichment analysis showed that these genes were mainly enriched in the protein digestion and absorption, TGF-β signaling pathway, ECM-receptor interaction and PPAR signaling pathway (Fig. 4H).

**Fig. 5:**
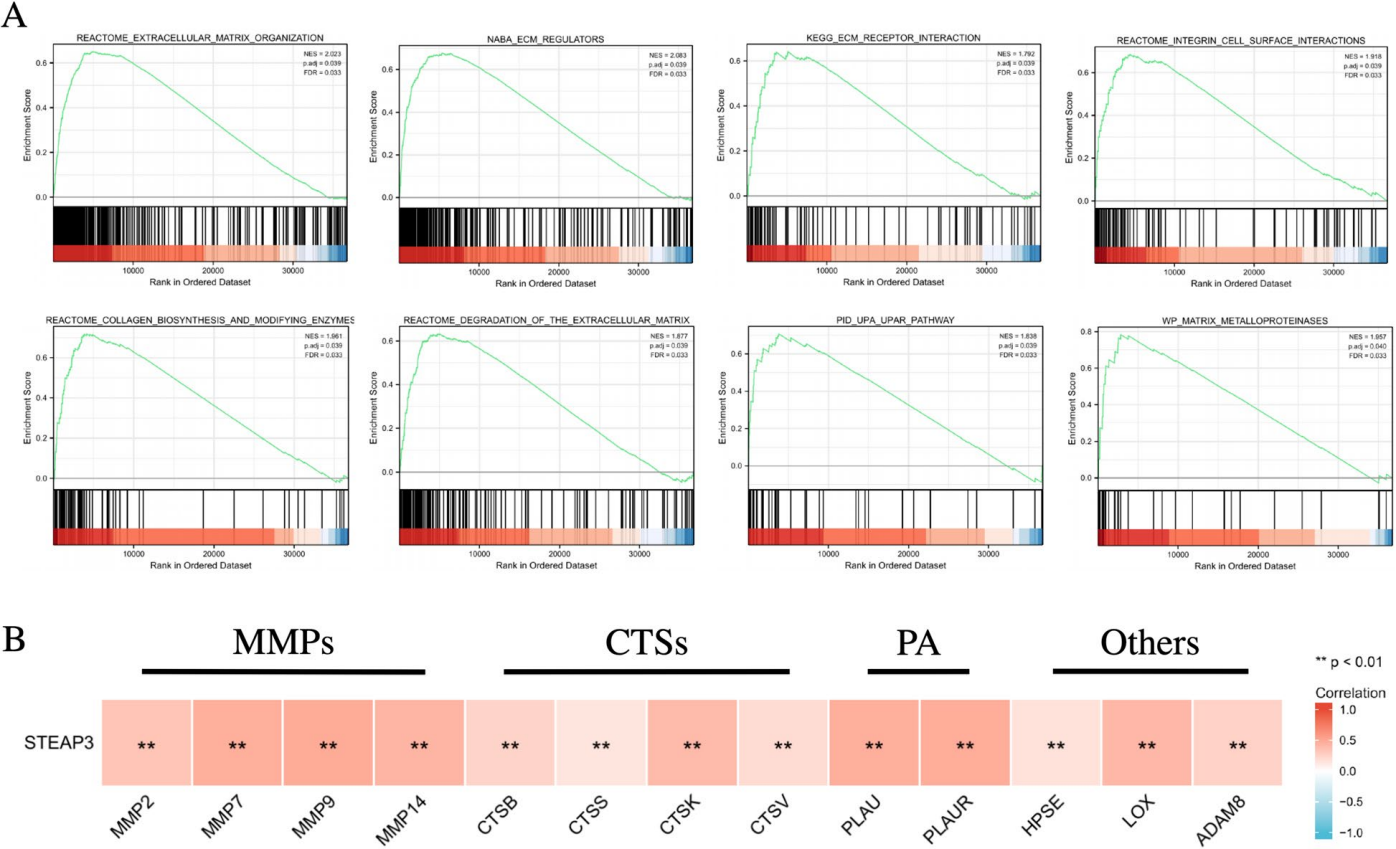
**A** GSEA of the expression profiles between the two groups. **B** Correlation analysis between STEAP3 and extracellular matrix degradation- and remodeling-related genes in ccRCC

GSEA analysis was performed to further explore the molecular mechanism of STEAP3 in the process of ccRCC. The results showed that extracellular matrix organization, ECM regulators, ECM receptor interaction, integrin cell surface interactions, collagen biosynthesis and modifying enzymes, degradation of the extracellular matrix, upa-upar pathway, matrix metalloproteinases were enriched in the high-expression group (Fig. 5A). Further analysis showed that the STEAP3 expression in ccRCC was significantly correlated with extracellular matrix degradation- and remodeling-related genes (Fig. 5B). The above results strongly suggested that STEAP3 may be involved in the regulation of tumor extracellular matrix in ccRCC.

### Correlation between the STEAP3 expression and the immune infiltration in ccRCC

As a main component of the tumor microenvironment, the extracellular matrix can significantly influence tumor progression and immune cell infiltration. Since the ECM may be regulated by STEAP3 in the process of ccRCC, we next explored the effect of STEAP3 on immune cell infiltration.

**Fig. 6:**
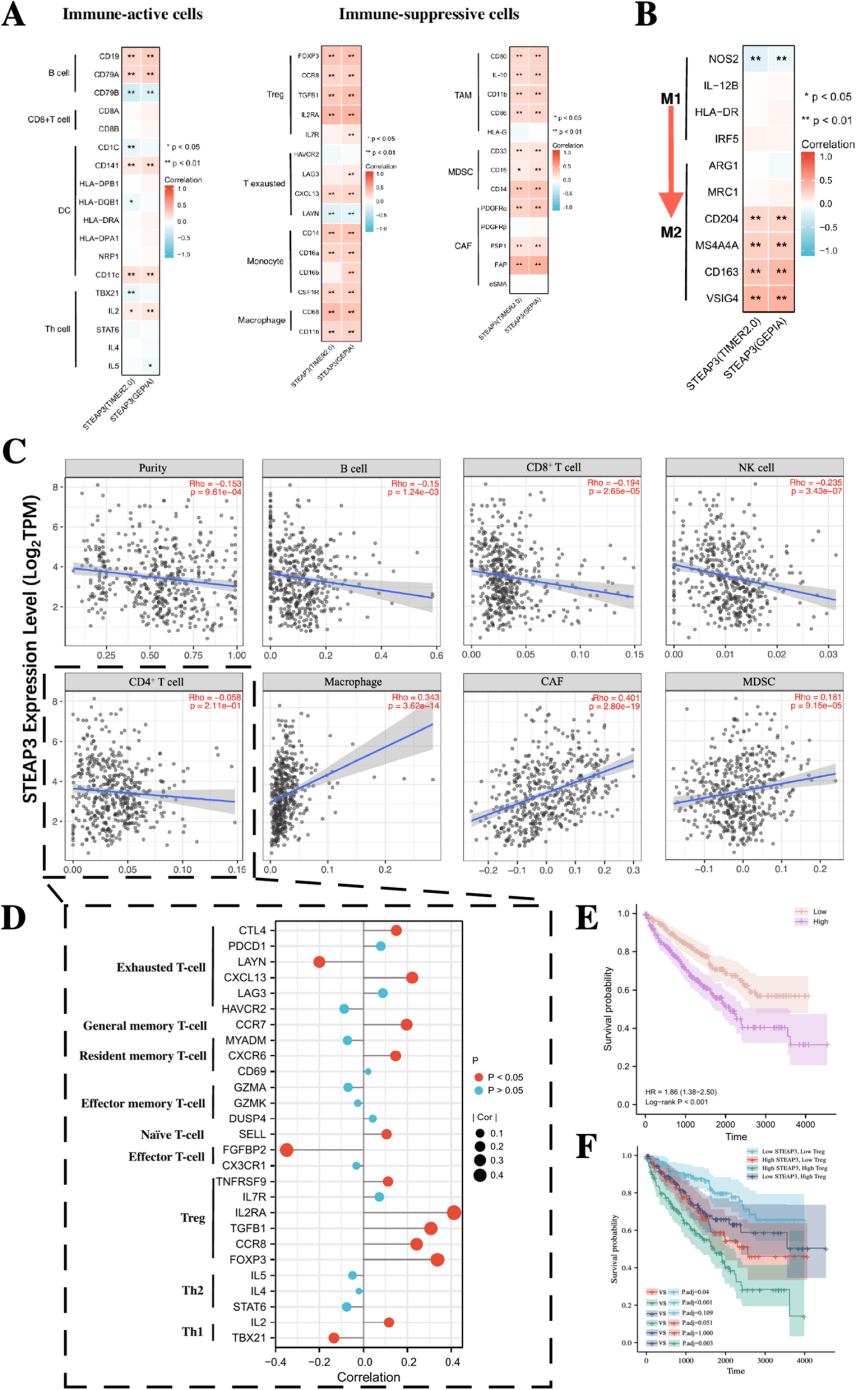
Overexpression of STEAP3 shapes an immune-suppressive tumor microenvironment. **A** Correlations between STEAP3 and the immune cell markers. **B** STEAP3 expression correlates with macrophages polarization. **C** The correlation between STEAP3 expression and the infiltrating levels of different immune cells. **D** Correlation of STEAP3 expression with markers of different CD4 + T cell subsets. **E** K-M survival analysis of the infiltrating levels of Tregs. **F** K-M survival analysis of STEAP3 expression combined with the infiltrating levels of Tregs. *p < 0.05; **p < 0.01; ***p < 0.001

We first explored the correlation between STEAP3 and immune cell markers that are widely used to characterize immune cells (Table 3). As shown in Fig. 6A, the expression level of STEAP3 was positively correlated with the markers of multiple immune-suppressive cells including Treg, T exhausted cell, monocyte, macrophage, tumor-associated macrophage (TAM), MDSC and tumor-associated fibroblasts (CAF). Besides, we also found that the STEAP3 expression was positively correlated with the phenotype of M2-macrophages (Fig. 6B). This suggested that high STEAP3 expression in ccRCC may contribute to the polarization of macrophages to the anti-inflammatory M2-macrophages, thereby promoting tumor progression.

**Table 3:** Correlations between STEAP3 and markers of immune cells

Cell Type	Markers	TIMER2.0				GEPIA	
		Correlation	p	Correlation^#^	*p* ^#^	Correlation	*p*
B cell	CD19	0.29	***	0.24	***	0.29	***
	CD79A	0.27	***	0.22	***	0.27	***
	CD79B	-0.23	***	-0.27	***	-0.21	***
CD8 + T cell	CD8A	0.08	0.06	0.06	0.17	0.079	0.07
	CD8B	0.05	0.22	0.02	0.63	0.044	0.31
DC	CD1C	-0.08	0.04	-0.15	**	-0.09	0.05
	CD141	0.17	***	0.13	**	0.18	***
	HLA-DPB1	0.04	0.39	-0.01	0.87	0.04	0.32
	HLA-DQB1	-0.07	0.13	-0.11	*	-0.05	0.30
	HLA-DRA	0.05	0.22	0.02	0.67	0.06	0.15
	HLA-DPA1	0.04	0.31	-0.01	0.92	0.05	0.23
	NRP1	0.02	0.70	-0.03	0.55	0.03	0.45
	ITGAX	0.18	***	0.19	***	0.17	***
Monocyte	CD14a	0.33	***	0.31	***	0.33	***
	CD16a	0.27	***	0.24	***	0.27	***
	CD16b	0.05	0.21	0.06	0.18	0.25	***
	CSF1R	0.28	***	0.24	***	0.27	***
TAM	CD80	0.23	***	0.22	***	0.25	***
	CD11b	0.25	***	0.23	***	0.25	***
	IL-10	0.23	***	0.21	***	0.25	***
	CD86	0.23	***	0.21	***	0.23	***
	HLA-G	-0.05	0.23	-0.09	0.06	-0.02	0.73
Macrophage	CD68	0.38	***	0.39	***	0.33	***
	CD11b	0.25	***	0.23	***	0.25	***
M1	NOS2	-0.15	***	-0.20	***	-0.12	**
	IL-12B	0.04	0.35	0.01	0.81	0.04	0.42
	HLA-DR	0.05	0.22	0.02	0.67	0.06	0.15
	IRF5	0.06	0.14	0.07	0.14	0.06	0.19
M2	ARG1	-0.02	0.59	0.01	0.90	-0.07	0.10
	MRC1	0.05	0.24	0.04	0.45	0.07	0.11
	CD204	0.25	***	0.24	***	0.25	***
	MS4A4A	0.30	***	0.27	***	0.3	***
	CD163	0.34	***	0.33	***	0.41	***
	VSIG4	0.43	***	0.43	***	0.44	***
MDSC	CD11b	0.25	***	0.23	***	0.25	***
	CD33	0.17	***	0.14	***	0.16	***
CAF	PDGFRa	0.29	***	0.25	***	0.31	***
	PDGFRb	0.01	0.78	-0.03	0.47	0.03	0.44
	FSP1	0.16	***	0.16	***	0.18	***
	FAP	0.47	***	0.43	***	0.45	***
	aSMA	0.03	0.50	-0.01	0.80	-0.01	0.90
Th cell	TBX21	-0.02	0.06	-0.13	*	-0.07	0.10
	IL2	0.15	***	0.12	*	0.15	***
	STAT6	-0.08	0.08	-0.08	0.10	-0.02	0.59
	IL4	-0.01	0.81	-0.02	0.65	-0.05	0.22
	IL5	-0.05	0.23	-0.05	0.28	-0.10	*
Treg	FOXP3	0.35	***	0.34	***	0.35	***
	CCR8	0.27	***	0.24	***	0.26	***
	TGFB1	0.35	***	0.31	***	0.36	***
	IL2RA	0.41	***	0.41	***	0.43	***
	IL7R	0.13	**	0.07	0.12	0.13	**
T exausted	HAVCR2 LAG3	-0.07 0.14	0.11 ***	-0.09 0.09	0.06 0.06	-0.05 0.12	0.25 **
	CXCL13	0.25	***	0.22	***	0.24	***
	LAYN	-0.18	***	-0.20	***	-0.16	***
T memory	CCR7	0.21	***	0.20	***	0.22	***
	MYADM	-0.08	0.07	-0.07	0.12	-0.06	0.20
	CXCR6	0.19	***	0.15	**	0.19	***
	CD69	0.07	0.10	0.02	0.65	0.08	0.09
	GZMA	0.00	0.93	-0.07	0.13	0.00	0.91
	GZMK	0.03	0.48	-0.03	0.57	0.02	0.59
	DUSP4	0.07	0.09	0.04	0.36	0.06	0.17
T effector	FGFBP2	-0.31	***	-0.35	***	-0.28	***
	CX3CR1	0.02	0.69	-0.03	0.48	0.03	0.44
T naive	SELL	0.16	***	0.11	*	0.17	***

To further confirm whether STEAP3 expression can affect the infiltration of immune cells in ccRCC, we explored the correlation between STEAP3 expression and immune infiltration in the TIMER2.0 database. The results showed that the abundance of B cells (*R* = -0.15, *p* = 0.001), CD8 + T cells (*R* = -0.194, *p* < 0.001) and NK cells (*R* = -0.235, *p* < 0.001) were negatively correlated with the STEAP3 expression; while the abundance of macrophages (*R* = 0.343, *p* < 0.001), CAFs (*R* = 0.401, *p* < 0.001) and MDSCs (*R* = 0.181, *p* < 0.001) were positively correlated with the STEAP3 expression (Fig. 6C). Given the STEAP3 expression was not significantly correlated with the abundance of CD4 + T cells, we further analyzed the correlation between the markers of different CD4 + T cell subsets and STEAP3 expression. According to Fig. 6D, we found that the effector T-cell marker (FDFBP2) was significantly negatively correlated with STEAP3, while the markers of Tregs were positively correlated with STEAP3. This suggested that the STEAP3 may be positively correlated with the abundance of immune-suppressive CD4 + T cell subsets (mainly Tregs) and negatively correlated with the abundance of immune-active subsets. Besides, a higher abundance of Tregs was found to be associated with poorer survival (Fig. 6E), and ccRCC patients with higher STEAP3 expression and higher abundance of Tregs seemed to have the worst prognosis (Fig. 6F). The above results suggested that high STEAP3 expression may mediate the formation of an immune-suppressive tumor microenvironment, thereby helping ccRCC evade recognition and clearance of immune cells.

### The correlation of Immune checkpoints and the sensitivity of targeted drugs

**Fig. 7:**
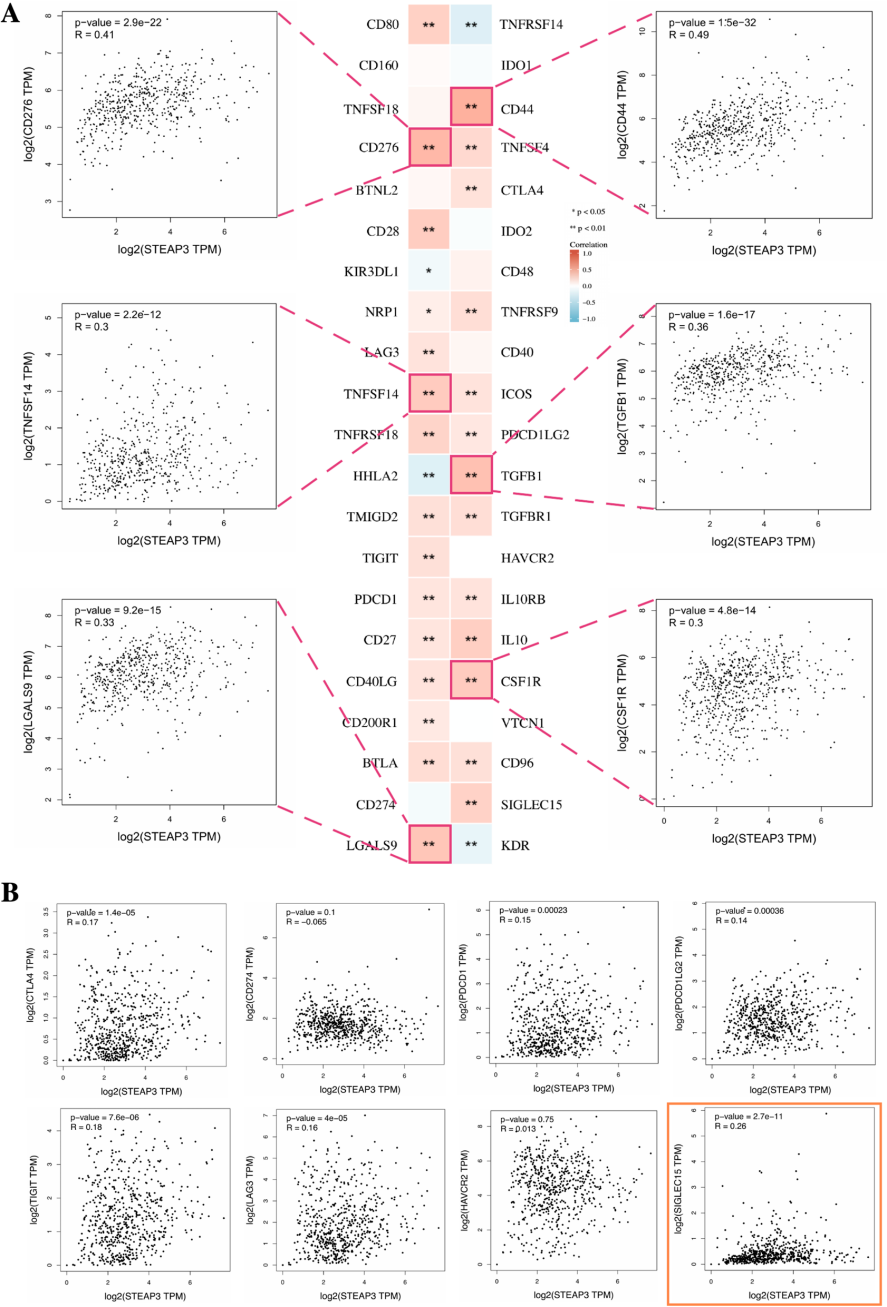
The correlation between STEAP3 and immune checkpoints. *p < 0.05; **p < 0.01; ***p < 0.001

Since ccRCC is insensitive to radiotherapy and chemotherapy, precise and effective treatment for it is still a major clinical challenge. To provide clinicians with effective immune and targeted therapy options for ccRCC. We comprehensively evaluated the correlation between STEAP3 expression and immune checkpoints, as well as the correlation between STEAP3 expression and the sensitivity of targeted drug. As shown in Fig. 7A, a total of 6 immune checkpoints were found to be significantly positive correlation (|R|> 0.3 and *p* < 0.05) with STEAP3 expression, including CD276, CD44, TNFSF14, TGFB1, LGALS9, CSF1R, indicating that targeting these six immune checkpoints may be an effective approach to treat STEAP3 high-expression patients. Besides, for classical immune checkpoints (CTLA4, PD-1, PD-L1, PD-L2, TIGIT, LAG3, HAVCR2 and SIGLEC15), we found a strong positive correlation between STEAP3 and SIGLEC15, indicating that the inhibitor against SIGLEC15 may also be effective for STEAP3 high-expression patients (Fig. 7B).

**Fig. 8:**
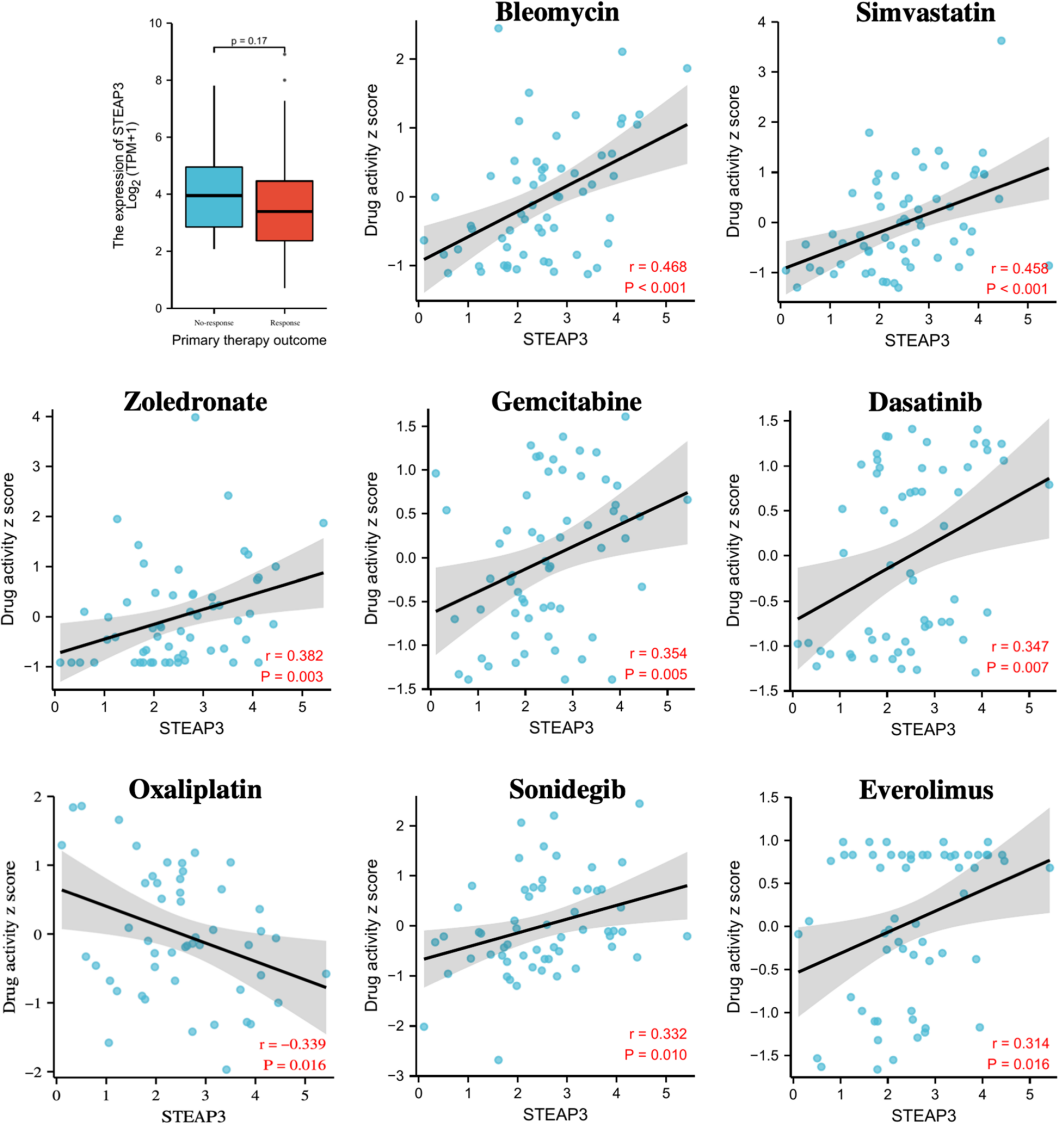
The correlation between STEAP3 expression and the sensitivity of targeted drugs. *p < 0.05; **p < 0.01; ***p < 0.001

Based on the response of ccRCC patients to targeted therapy, we investigated the STEAP3 expression between responders (SD, PR, CR) and non-responders (PD). As shown in Fig. 8, STEAP3 was found to be relatively higher in non-responders, so screening out drugs that can effectively target these patients may have some clinical value. By analyzing the data in the CellMiner database, we finally screened eight targeted drugs with |R|> 0.3 and *p* < 0.05. Bleomycin, Simvastatin, Zoledronate, Gemcitabine, Dasatinib, Sonidegib and Everolimus appeared to be more beneficial in STEAP3 high-expression patients, while Oxaliplatin seemed to be more effective for STEAP3 low-expression patients (Fig. 8).

### Experimental verification

**Fig. 9:**
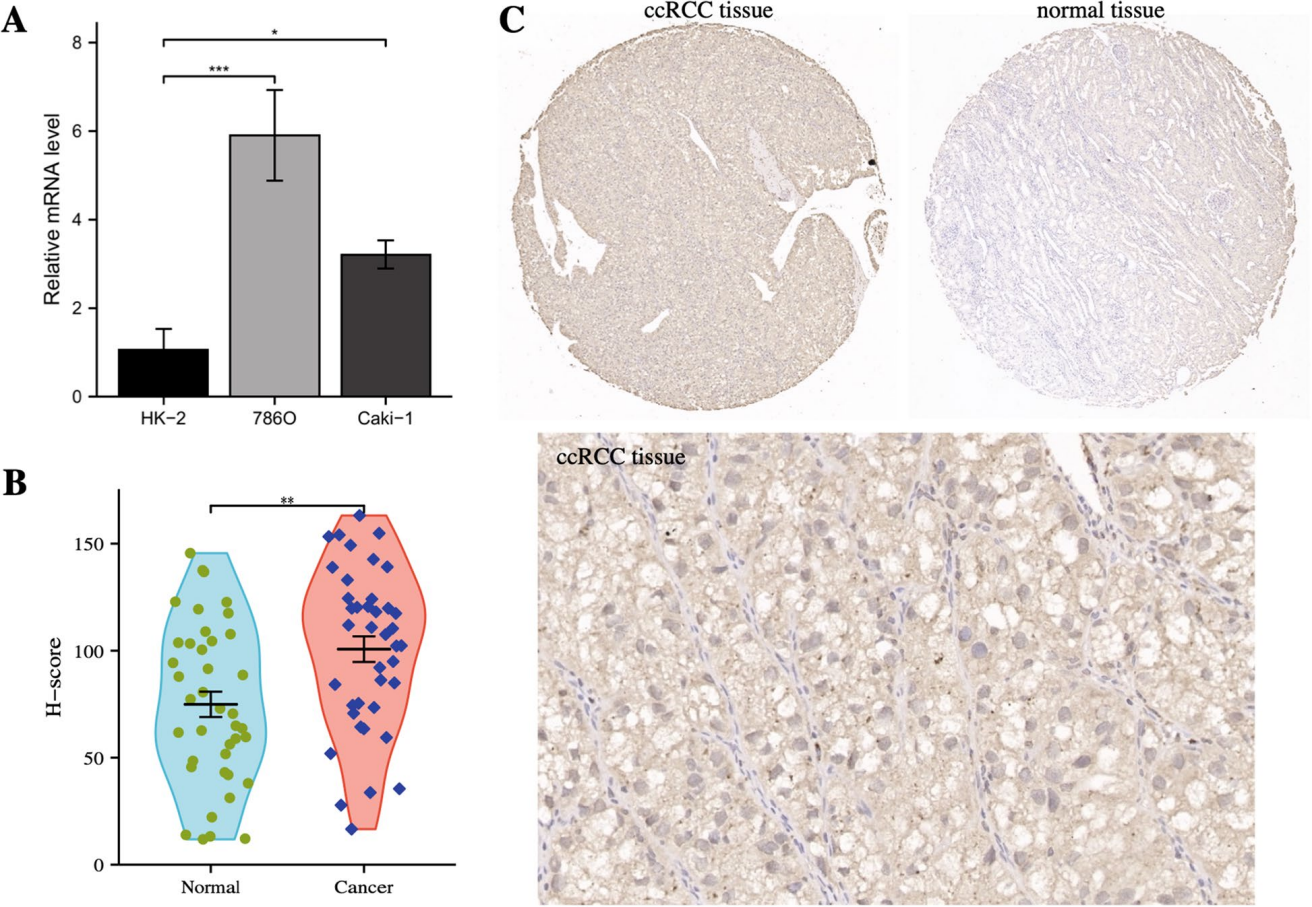
Experimental verification. **A**: the mRNA expression levels of STEAP3 between HK-2 cells, 786O cells and Caki-1 cells. **B**-**C**: Immunohistochemical staining for STEAP3 in ccRCC tissues and normal tissues

RT-qPCR was conducted to verifity the STEAP3 expression in HK-2 cells, 786O cells and Caki-1 cells. Compared with HK-2 cells, the STEAP3 expression were significantly increased in 786O cells and Caki-1 cells (Fig. 9A), which supported our previous analysis results. IHC staining showed the protein expression of STEAP3 was also increased in ccRCC tissues compared with normal kidney tissue (Fig. 9B-C).

## Discussion

In recent years, studies have shown that iron metabolism dysregulation is a common phenomenon in many cancers (20). The excess iron can not only promote tumorigenesis, but also help tumor proliferation and distant metastasis through its pro-oxidative effects (13). As a key enzyme for reducing Fe3 + to Fe2 + in endosomes, STEAP3 has been extensively studied for its role in the process of various tumors (21, 22). However, there is still no research to explore the relationship between STEAP3 and the development of ccRCC.

In this study, we conducted a detailed investigation to clarify the underlying mechanism and specific role of STEAP3 in ccRCC. By integrating information from multiple public databases, we found that STEAP3 was abnormally expressed in various tumor tissues, and its expression was significantly correlated with the prognosis of cancer patients. As for ccRCC, the mRNA and protein expression levels of STEAP3 were significantly up-regulated in tumor tissues, and STEAP3 expression was negatively correlated with the prognosis of ccRCC. This is highly consistent with a recent research by Rocha et al. (23), in which they used the Oncomine and CBioPortal databases to conduct a comprehensive analysis of the expression and prognosis of STEAP family members in human cancers. Independent prognostic analysis showed high STEAP3 expression, advanced age, low hemoglobin level, lesion in the left kidney, high pathological grade and clinical stage were prognostic risk factors for ccRCC. By integrating the above prognostic risk factors, we constructed a nomogram, which can provide a reference for clinicians to evaluate prognosis.

The functional enrichment analysis showed that compared with STEAP3 low-expression ccRCC, STEAP3 high-expression ccRCC had obvious degradation and remodeling of tumor extracellular matrix. The immune infiltration analysis showed that STEAP3 high-expression ccRCC had a higher abundance of immune-suppressive cells and a lower abundance of immune-effector cells. These results suggested that STEAP3 may be involved in the regulation of the tumor microenvironment of ccRCC. It is well known that STEAP3 can reduce Fe3 + to Fe2 + in endosomes, and Fe2 + will generate a large number of reactive oxygen species (ROS) in the process of various redox reactions (24). ROS can not only stimulate malignant transformation of normal cells, but also is essential for maintaining tumor proliferation and metastasis (25). Recently, the regulatory effect of ROS on the tumor microenvironment has been extensively studied.

The extracellular matrix is a complex protein network that provides mechanical and structural support for tumors (26). Studies have shown that STEAP3 can promote the remodeling and deposition of extracellular matrix in an oxidative stress-dependent manner (generation of ROS) during wound healing in diabetic patients (27). In many tumors, ROS has also been proved to degrade and remodel the tumor extracellular matrix through TGF-β1, hypoxia, NOX4 and other mechanisms (28, 29). Extracellular matrix remodeling occurs dynamically at different stages of tumors, which can promote tumor proliferation, invasion, and metastasis. Studies have shown that extracellular matrix remodeling can regulate pathways such as integrin-mediated signaling, TGF-β/STAT3 signaling, and DDR1/STAT3 signaling to activate multiple signaling pathways to promote tumor survival and proliferation (30–32). The degradation of matrix components by various enzymes (such as proteolytic enzymes, matrix metalloproteinases, etc.) and the transformation and arrangement of collagen fibers in the matrix create favorable conditions for tumor cell invasion and metastasis (33, 34). In addition, extracellular matrix remodeling has also been found to influence the infiltration of immune cells in the tumor microenvironment. On the one hand, it has been shown that ECM-related proteins are abundant and regularly arranged in the periphery of the tumor, while less and disordered in the center. This distribution pattern can hinder the motility and infiltration of T cells, thereby facilitating tumor evasion of immune clearance (35). Rossi et al. showed that matrix metalloproteinases (MMPs) can drive the shedding of adhesion molecules ICAM-1 and B7-H6 on the surface of NK cells and T cells, thereby reducing their cytotoxicity to tumor cells (36). On the other hand, hyaluronic acid (HA) can be degraded into fragments of different sizes during extracellular matrix remodeling, and it was found that HA fragments with large molecular weight can bind to CD44 on Treg to up-regulate the expression of Foxp3 and further enhance the immunosuppressive capacity of Treg (37). Changes in ECM composition and the release of cytokines can also increase the infiltration of TAMs and the transformation of myofibroblasts, which contributes to strengthening the tumor stroma and promoting immunosuppression (38, 39).

In addition to extracellular matrix remodeling, ROS has also been found to directly affect tumor immunity. For immune-suppressive cells, ROS can promote the differentiation of immature CD4 + T cells into Tregs and enhance the immunosuppressive function of Tregs (40). Besides, ROS induces the differentiation of myofibroblast into CAFs by up-regulating CXCL12 and down-regulating CAV1 (41) and promotes the activation of CAFs by inducing the expression of TGF-β1, NF-κB and STAT in CAFs (42). For TAMs, studies have found that elevated ROS in the tumor microenvironment contributes to the differentiation of TAMs into the M2 subtype (43), while the elimination of ROS can inhibit the polarization of M2 macrophages through the STAT3 signaling pathway (44). Ohl et al. found that ROS plays an important role in maintaining the undifferentiated state of MDSCs, and clearance of ROS can lead to the differentiation of MDSCs into macrophages and DCs in various cancer models (45). For immune-effector cells, studies have shown that elevated ROS can induce the weakening and exhaustion of T cells through chronic oxidative stress, thereby inhibiting their activation, proliferation, and the function of anti-tumor (46, 47). In NK cells, increased ROS can consistently activate the mTOR/Drp1 pathway, leading to mitochondrial fission and subsequent apoptosis, thereby inhibiting NK cell activity and tumor-killing capacity (48). The role of ROS on B cells is complex. Studies have shown that low concentrations of ROS can promote the activation and proliferation of B cells, while high concentrations of ROS can prevent B cell survival through autophagy pathways (49, 50). In the present study, we found that STEAP3 high-expression ccRCC holds obvious remodeling of the extracellular matrix, and its immune infiltration pattern was significantly different from STEAP3 low-expression ccRCC. Therefore, we hypothesized that in STEAP3 high-expression ccRCC, the redox reaction involving Fe2 + produced a large number of ROS, and these ROS can promote the process of ccRCC by regulating the tumor microenvironment.

To provide guidance for clinical treatment of ccRCC, we also explored the correlation of Immune checkpoints and the sensitivity of targeted drugs. The results of immune checkpoints correlation analysis showed that CD44, CD276, TGFB1, LGALS9, and CSF1R had the highest correlation with STEAP3. Analysis of classical immune checkpoints showed that STEAP3 was highly correlated with SIGLEC15, suggesting that ccRCC patients with high STEAP3 expression may benefit from the inhibitor against SIGLEC15. Targeted drug sensitivity analysis identified eight drugs with IC50 significantly correlated with STEAP3, among which B Bleomycin, Simvastatin, Zoledronate, Gemcitabine, Dasatinib, Sonidegib and Everolimus may be more effective for STEAP3 high-expression patients, while Oxaliplatin may be the first choice for STEAP3 low-expression patients.

In conclusion, in the present study, we found that the expression of STEAP3 was significantly up-regulated in ccRCC tissues, and STEAP3 expression was negatively correlated with the prognosis of ccRCC. Furthermore, high STEAP3 expression seemed to regulate the tumor microenvironment of ccRCC through an oxidative stress-dependent manner. Therefore, we suggested that the STEAP3 (an iron metabolism-related gene) may play an important role in the process of ccRCC. It may serve as a prognostic biomarker for ccRCC and targeting STEAP3 expression may be an effective strategy for the treatment of ccRCC in the future.

## Supplementary Information

**Additional file 1: Table S1: **Gene ontology (GO) enrichment analysis of the STEAP3-related genes.

**Additional file 2: Table S2:** Kyoto Encyclopedia of Genes and Genomes (KEGG) pathways analysis of the STEAP3-related genes.

## Data Availability

The authors confirm that the data and materials supporting the findings of this study are available on the online database and the supplementary materials.

## Abbreviations

ccRCC: Clear cell renal cell carcinoma
ICIs: Immune checkpoint inhibitors
CTLA-4: Cytotoxic T lymphocyte-associated antigen 4
PD-1/PD-L1: Programmed death 1/programmed death ligand 1
OS: Overall survival
DSS: Disease-free survival
PFI: Progression-free interval
PPI: Protein–protein interaction
BP: Biological process
CC: Cell composition
MF: Molecular function
