# Supplementary material for: STEAP3 can predict the prognosis and shape the tumor microenvironment of clear cell renal cell carcinoma
Source: BMC Cancer. 2022 Nov 23;22:1204. doi: 10.1186/s12885-022-10313-z (PMC9686107; doi:10.1186/s12885-022-10313-z)
Supplement: Supplementary file 1 — Additional file 1: Table S1: Gene ontology (GO) enrichment analysis of the STEAP3-related genes. [file 12885_2022_10313_MOESM1_ESM.docx]

**TableS1:** Gene ontology (GO) enrichment analysis of the STEAP3-related genes.

| Ontology | Term | P value | Count |
| --- | --- | --- | --- |
| BP | extracellular structure organization | 1.776E-27 | 66 |
| BP | extracellular matrix organization | 7.6394E-27 | 61 |
| BP | negative regulation of peptidase activity | 1.3885E-11 | 33 |
| BP | acute inflammatory response | 9.8821E-14 | 33 |
| BP | connective tissue development | 1.878E-10 | 32 |
| BP | negative regulation of endopeptidase activity | 3.9494E-10 | 30 |
| BP | cartilage development | 2.2743E-11 | 29 |
| BP | chondrocyte differentiation | 4.0186E-11 | 22 |
| BP | acute-phase response | 2.909E-14 | 17 |
| BP | collagen fibril organization | 7.4421E-11 | 15 |
| CC | collagen-containing extracellular matrix | 6.5317E-30 | 68 |
| CC | endoplasmic reticulum lumen | 2.1862E-13 | 39 |
| CC | collagen trimer | 2.5613E-11 | 19 |
| CC | basement membrane | 1.3016E-10 | 19 |
| CC | extracellular matrix component | 4.1714E-10 | 14 |
| CC | blood microparticle | 4.6066E-08 | 20 |
| CC | high-density lipoprotein particle | 6.1287E-08 | 9 |
| CC | fibrillar collagen trimer | 4.1096E-07 | 6 |
| CC | banded collagen fibril | 4.1096E-07 | 6 |
| CC | complex of collagen trimers | 1.1606E-06 | 7 |
| MF | peptidase regulator activity | 3.0094E-11 | 30 |
| MF | peptidase inhibitor activity | 4.6367E-11 | 27 |
| MF | endopeptidase regulator activity | 2.4905E-10 | 26 |
| MF | endopeptidase inhibitor activity | 5.5236E-10 | 25 |
| MF | serine-type endopeptidase inhibitor activity | 1.2319E-09 | 18 |
| MF | glycosaminoglycan binding | 8.4051E-09 | 27 |
| MF | collagen binding | 2.6695E-08 | 14 |
| MF | heparin binding | 3.4056E-08 | 22 |
| MF | enzyme inhibitor activity | 8.338E-08 | 34 |
| MF | passive transmembrane transporter activity | 3.9436E-07 | 37 |
