# Supplementary material for: STEAP3 can predict the prognosis and shape the tumor microenvironment of clear cell renal cell carcinoma
Source: BMC Cancer. 2022 Nov 23;22:1204. doi: 10.1186/s12885-022-10313-z (PMC9686107; doi:10.1186/s12885-022-10313-z)
Supplement: Supplementary file 2 — Additional file 2: Table S2: Kyoto Encyclopedia of Genes and Genomes (KEGG) pathways analysis of the STEAP3-related genes. [file 12885_2022_10313_MOESM2_ESM.docx]

**TableS2:** Kyoto Encyclopedia of Genes and Genomes (KEGG) pathways analysis of the STEAP3-related genes.

| Id | Term | P value | Count |
| --- | --- | --- | --- |
| hsa04610 | Complement and coagulation cascades | 6.8228E-12 | 19 |
| hsa05202 | Transcriptional misregulation in cancer | 0.00239741 | 14 |
| hsa04974 | Protein digestion and absorption | 2.8966E-06 | 14 |
| hsa05150 | Staphylococcus aureus infection | 0.00016683 | 11 |
| hsa04061 | Viral protein interaction with cytokine and cytokine receptor | 0.0009799 | 10 |
| hsa04978 | Mineral absorption | 1.0762E-05 | 10 |
| hsa04933 | AGE-RAGE signaling pathway in diabetic complications | 0.00359976 | 9 |
| hsa04350 | TGF-beta signaling pathway | 0.00236102 | 9 |
| hsa04512 | ECM-receptor interaction | 0.00148799 | 9 |
| hsa03320 | PPAR signaling pathway | 0.00265413 | 8 |
